# Supplementary material for: miR‐29 contributes to normal endothelial function and can restore it in cardiometabolic disorders
Source: EMBO Mol Med. 2018 Jan 26;10(3):e8046. doi: 10.15252/emmm.201708046 (PMC5840545; doi:10.15252/emmm.201708046)
Supplement: Supplementary file 1 — Appendix [file EMMM-10-e8046-s001.pdf]

APPENDIX

miR-29 Contributes to Normal Endothelial Function and can Restore it in Cardiometabolic Disorders

Michael E. Widlansky[1,*,#], David M. Jensen[2,#], Jingli Wang[1], Yong Liu[2], Aron M. Geurts[2], Alison J. Kriegel[2], Pengyuan Liu[2], Rong Ying[1], Guangyuan Zhang[2], Marc Casati[2], Chen Chu[2], Mobin Malik[1], Amberly Branum[1], Michael J. Tanner[1], Kristie Usa[2], Sudhi Tyagi[1], Mingyu Liang[2,*]

[1]Department of Medicine, Division of Cardiovascular Medicine, [2]Center of Systems Molecular Medicine, Department of Physiology, Medical College of Wisconsin, Milwaukee, WI, USA

[#]co-first author

*Correspondence:

Michael Widlansky, mwidlans@mcw.edu

Mingyu Liang, mliang@mcw.edu

Appendix

Appendix Materials and Methods

Appendix Tables S1-S3

Table S1. Demographic, clinical, and medication information and in vivo vascular function for the 38 subjects with resistance arterioles that underwent miRNA-Seq profiling

Table S2. Known human miRNAs in the top 30 miRNAs most significantly differentially expressed in gluteal arterioles of DM compared to Non-DM subjects.

Table S3. Demographic, clinical, and medication information for the 70 subjects with resistance arterioles used in miRNA mimic or anti-miR treatment experiments.

Table S4. Exact p values.

Appendix Figures S1 to S6

Figure S1. Real-time PCR verification of small RNA deep sequencing results.

Figure S2. Confirmation of miR-29 mimic transfection in human gluteal arterioles.

Figure S3. Spermine NONOate, a nitric oxide (NO) donor, induced robust dilation in human arterioles after intraluminal treatment with anti-miR-29b-3p.

Figure S4. Abundance of miR-29b-3p in the EC elute from the aorta of *Mir29b-1/a*$^{-/-}$ rats.

Figure S5. The NO donor spermine NONOate induced robust dilation in arterioles from *Mir29b-1/a*$^{-/-}$ rats.

Figure S6. eNOS protein abundance in mesentery arteries was not changed in *Mir29b-1/a*$^{-/-}$ rats.

Figure S7. RNA-seq data from gluteal arterioles correctly clustered *Mir29b-1/a*$^{-/-}$ rats (KO) and wild-type littermates (WT) separately.

Figure S8. Abundance of Gap43 mRNA in the carotid artery of *Mir29b-1/a*$^{-/-}$ rats.

Appendix References

Appendix Materials and Methods

Brachial Artery Reactivity Testing, and Measurement of In Vitro Endothelial Function by

In vivo measurements of endothelial function were performed by flow mediated dilation (FMD) as previously described.(Babar et al, 2011; Kizhakekuttu et al, 2010; Kizhakekuttu et al, 2012; Wang et al, 2012) Brachial artery images were captured using a high resolution ultrasound. Images were recorded prior to and following analysis and blood pressure cuff inflation. Brachial diameters, flow mediated dilatation (FMD%), nitroglycerin-mediated dilatation (NMD%), baseline and post-deflation (hyperemic) peak flow velocity, and baseline and post-deflation peak shear stress in the brachial artery were measured as previously described.(Kizhakekuttu et al, 2010)

Gluteal Adipose Pad Biopsy and Harvesting of Resistance Arterioles,

Following local sterilization of the upper outer gluteal quadrant on the subjects non-dominant side with either iodine or chlorhexidine followed by local administration of 2% lidocaine, a ~1-1.5 cm incision was made in the upper out gluteal quadrant. Subcutaneous adipose samples measuring on average 1.5 x 1.5 x 1.5 cm were removed by sharp dissection. Following hemostasis, wounds were closed with a single absorbable suture and further opposed with SteriStrips (3M, St. Paul, MN). Arterioles (50 to 120 µm in diameter) were isolated from the gluteal biopsy in cold (4°C) HEPES buffered saline. Fat and connective tissues around the arterioles were removed before the arterioles were used for experiments.

Videomicroscopy for Non-Transfected Arterioles

Endothelium-dependent vasodilation to acetylcholine was measured in arterioles in the initial cohort by videomicroscopy as we described previously. (Dharmashankar et al, 2012; Kizhakekuttu et al, 2012; Suboc et al, 2013; Wang et al, 2012) Briefly, isolated arterioles were transferred to an organ bath and cannulated with tapered glass micropipettes filled with cold Krebs buffer. Vessel ends were secured with 10-0 nylon Ethilon monofilament suture (22 µm diameter; Look, Norwell, MA), Arterioles were subsequently transferred to the stage of an inverted microscope attached to a video camera, video monitor, and a video-measuring device (model VIA-100; Boeckeler). Vessels were imaged at a magnification of 200x. Before testing, arterioles were equilibrated at an intraluminal pressure of 20 mmHg followed by 60 mmHg (30 minutes at each pressure). Arterioles were continuously superfused with Krebs buffer bubbled with a gas mixture of 21% O_2-5% CO_2-74% N_2. To test endothelium-dependent vasodilation, acetylcholine was used at final concentrations of 10^{-10} to 10^{-5} mol/L in the chamber after the arteriole was pre-constricted to 50% to 70% of maximum diameter in resting condition by enothelin-1. Endothelium-independent vasodilation was examined by adding papaverine (2 x 10^{-4} mol/L in final concentration). Vasodilatory response to acetylcholine is reported as % of maximum dilation, which was defined as (vessel diameter – pre-constricted diameter)/(maximum diameter – pre-constricted diameter), where maximum diameter was the resting diameter or the diameter following the addition of papaverine, whichever was greater. In some arterioles, after recoding basal levels of vasodilation, the above protocol was repeated with the addition of L-NAME (100 µM for 30 minutes) in the chamber to determine the eNOS dependence of the response.

Nitric oxide (NO) measurement

NO levels were measured in intact arterioles using 4,5-Diaminofluorescein diacetate (DAF-2 DA) fluorescent dye with appropriate controls as recommended by Griendling et al.(Griendling et al, 2016; Kojima et al, 1998) Insufficient amounts of tissue are present to make reliable nitrate/nitrite measurements using the Griess reaction. Gluteal arterioles isolated from rats or humans were incubated with 5 nM of DAF-2 DA (EMD Millipore, MA, USA) in PBS buffer at 37°C for 30 minutes. After three

washes, the arterioles were mounted on a slide with fluorescent mounting medium (Dako, CA, USA) and fluorescence generated from DAF-2 DA was detected under a fluorescence microscope (Nikon E55i, Tokyo, Japan) with cube at the position of FITC (Ex/Em: 465-495/515-555 nm). The vessel images were taken by image software NIS-Elements D 3.2, and the fluorescence intensity was analyzed by using MetaMorph 6.1 software (Universal Imaging, West Chester, PA). Data was expressed with arbitrary unit.

NO production in cultured cells was measured by the DAF2 method and nitrite and nitrate analysis. For the DAF2 measurement, culture media was removed and replaced with 200 µl of HBSS (ThermoFisher Scientific) with 100 µM L-arginine (Sigma-Aldrich) after 2 washes with HBSS. DAF-2 DA (5 nM) was added to each well and the plate was incubated for 30 minutes at 37°C in the dark. Cells were rinsed twice with HBSS and 200 µl of HBSS was pipetted into each well. To half of the wells for each treatment, 1 mM L-NAME (Sigma-Aldrich) was added. Following 10 minutes of incubation at 37°C in the dark, 5 µM calcium ionophore A23187 (Sigma-Aldrich) was added to half of the wells and the cells were incubated for another 5 minutes at 37°C in the dark. Fluorescence was measured using a SpectraFluor plate reader (Tecan, Switzerland) using 485 nm excitation and 535 nm emission filters. For nitrite and nitrate measurement, cells were treated as described above and the culture supernatant was collected. The total concentration of nitrate and nitrite was analyzed using a Nitrate/Nitrite Fluorometric Assay Kit (Cayman Chemical, Cat # 780051) and read with a SpectraFluor plate reader (Tecan, Switzerland) using a 550 nm absorbance filter.

Small RNA Deep Sequencing and Data Analysis

Additional arterioles were obtained from biopsy samples from 38 subjects for RNA extraction and subsequent miRNA expression profiling by small RNA deep sequencing. We have previously validated this method for measuring miRNA expression in small amount of tissues.(Kriegel et al, 2015; Kriegel et al, 2013) Briefly, RNA was extracted using TRIzol. Small RNA libraries were prepared from each of the 38 individual samples using TruSeq Small RNA Sample Preparation Kit from Illumina largely following the vendor's instructions. Cluster generation and sequencing were done using TruSeq PE Cluster Kit v3-cBot-HS, TruSeq SBS kit v3-HS, and an HiSeq 2000 sequencer (Illumina). Detection of known and novel microRNAs from the deep sequencing data were described previously.(Kriegel et al, 2015; Kriegel et al, 2013) Briefly, the adapter sequences were first removed from the output reads by the tool cutadapt (http://code.google.com/p/cutadapt/). Sequences with low quality (base quality < 13) at both ends of reads were further trimmed and trimmed reads with 15-25 bases were retained using the FASTQC (http://www.bioinformatics.babraham.ac.uk/projects/fastqc/). The trimmed reads were then mapped against miRBase v18 using miRanalyzer.

Differential expression of microRNAs between diabetes and non-DM subjects were examined using deGPS R package (https://github.com/LL-LAB-MCW). deGPS is a recently developed tool for detecting differential expression in RNA-Seq data. It contains new normalization methods based on generalized Poisson distribution modeling of sequence count data, followed by permutation-based differential expression tests. Association of phenotypes with microRNA expression with or without the adjustment of diabetes status was examined using generalized linear models (GLMs) in R packages. Sex and age generally did not show significant association with microRNA levels in the current study and were, therefore, not included as cofounding factors in the GLMs. The Benjamini-Hochberg procedure was used for controlling false discovery rates in multiple comparisons.

Real-time PCR

Real-time PCR analysis of miRNAs was carried out with Taqman chemistry as we described previously.(Mladinov et al, 2013; Tian et al, 2008a) Primers and probes for miRNAs were obtained from Thermo Fisher Scientific. RNU6B was used as internal normalizer. Real-time PCR analysis of mRNA was performed using SYBR Green chemistry as we described previously.(Kriegel et al, 2012) Primer

sequences (5'-3') are TGCGGTGATTTTCCTGCAT and ATTGGGAGATACTGGGCACGGATGG for human LYPLA1, GGAAGAAGGCAGGGGAAGATAC and AGCTAGCCTGAATTTTGGTTGC for rat Gap43, GCATTTTTGGGAGGTGAGAGG and TTGACGGCAGCAGAGCAGA for rat PECAM-1, and GACATTACCCCACCTCTCACCA and GCTCCCGACCTGCATCTATCT for rat MYH11. 18S rRNA was used as internal normalizer.

Isolation of rat gluteal resistance arterioles and assessment of endothelial function

Rat gluteal arterioles (60 to 150 µm in diameter) were isolated in cold (4°C) HEPES buffered saline. After removing surrounding fat and connective tissues, the arteriole were transferred to an organ bath and cannulated with tapered glass micropipettes filled with physiological salt solution consisting of (in mM) 119 NaCl, 4.7 KCl, 1.6 CaCl2, 0.03 EDTA, 1.17 MgSO4, 24 NaHCO3, 1.18 KH2PO4, and 5 glucose. The organ bath was transferred to the stage of an inverted microscope attached to a video camera, video monitor, and a video-measuring device. The arteriole was allowed to equilibrate at an intraluminal pressure of 30 mmHg supplied by a reservoir system for 30 minutes and followed by 60 mmHg for another 30 minutes. Endothelium-dependent vasodilation was determined as described above for human gluteal arterioles except U46619 was used to pre-constrict the arteriole.(Ou et al, 2003)

Blood pressure measurement in conscious, freely moving rats

Chronic instrumentation for blood pressure measurement in conscious, freely moving rats was performed as we described previously.(Liu et al, 2010; Tian et al, 2009) Briefly, an arterial catheter was implanted into the left femoral artery and exteriorized from the back of the rat. Rats were allowed 1 week to recover before the initiation of daily blood pressure recording through the arterial catheter.

RNA-seq and data analysis

RNA-seq analysis was performed as we described previously.(Liu et al, 2014) Briefly, total RNA was extracted from isolated rat gluteal arterioles using TRIzol. Five µg of total RNA was used to prepare mRNA (cDNA) libraries using TruSeq RNA Sample Preparation Kit from Illumina, following the vendor's instructions. Cluster generation and sequencing were similar to that described under Small RNA Deep Sequencing. RNA-seq data was analyzed using an in-house analytical pipeline. Differential expression of transcripts was identified using false discover rate (FDR) < 0.05.

Cell culture and transfection

Human dermal microvascular endothelial cells (HMVEC-d) were obtained from Lonza (Switzerland) and cultured as we described previously.(Kriegel et al, 2015; Liang & Pietrusz, 2007) All cells used in experiments were between passage 3 and 8. Cells were transfected with miR-29b-3p mimic or scramble miRNA mimic (ThermoFisher Scientific) at a final concentration of 35 nM. Transfection was carried out using Lipofectamine 2000 following the manufacturer protocol (ThermoFisher Scientific). Cells were then incubated for 24 hours before being used for analysis. For the transfection with LYPLA1 expression constructs, EGM-2 MV Growth Medium (Lonza) was switched to Opti-MEM reduced serum media (ThermoFisher Scientific) and each well of a 96-well plate was transfected with 500 ng of either pCMV6-Entry mammalian vector (Origene, Cat. # PS100001) or LYPLA1 Human cDNA ORF Clone (Origene, Cat. # RC202029). Transfection was carried out using Lipofectamine 2000 following the manufacturer's protocol. Four hours later, media was replaced with EGM-MV Growth Medium and cells were transfected with miR-29b-3p mimic or scramble miRNA mimic at a final concentration of 35 nM. miRNA transfections were carried out using Lipofectamine RNAiMax (ThermoFisher Scientific) following the manufacturers protocol. Cells were analyzed 24 hours later.

Western blot

Western blot analysis was performed as we described previously.(Tian et al, 2008a; Tian et al, 2008b) Primary antibody for LYPLA1 was from Sigma Aldrich (SAB2101408, rabbit polyclonal) and used at 1:500 dilution. Secondary antibody was from Santa Cruz Biotechnology (sc-2004, goat anti-rabbbit IgG-HRP) and used at 1:5,000 dilution. Band intensity was normalized by Coomassie blue staining of the entire membrane.

3'-UTR Reporter Assay

3'-UTR reporter assay was performed as we described previously.(Liu et al, 2010; Mladinov et al, 2013) Primers used for cloning a segment of the 3'-UTR of human LYPLA1 mRNA from nucleotide position 29 to 1,579, relative to the stop codon, were (5'-3') aaatttACTAGTcaccagcatcattgtagtag and tatatAAGCTTttgctgatttgtagaagtga. Primers for generating a mutated or partially deleted human 3'-UTR construct were gaatgcatctttataaatgggggcgaattgataatggaaataatttag and ctaaattatttccattatcaattcgcccccatttataaagatgcattc, and gaatgcatctttataaatgggaattgataatggaaataatttagtaatgg and ccattactaaattatttccattatcaattcccatttataaagatgcattc, respectively. Primers for cloning a segment of the 3'-UTR of rat Lypla1 mRNA from nucleotide position 662 to 1,446, relative to the stop codon, were (5'-3') aaaaaACTAGTattgcctccctgtcataaaga and tatatAAGCTTttcaaagtaaagcaaaatagtaaa. Primers for generating a mutated or partially deleted rat 3'-UTR construct were ggaagcatctttgtaagcaggttctaactgatgtggggtc and gaccccacatcagttagaacctgcttacaaagatgcttcc, and ggaagcatctttgtaagcaaactgatgtggggtc and gaccccacatcagtttgcttacaaagatgcttcc, respectively.

Extraction of endothelium-enriched fractions from rat gluteal arterioles and aorta

Extraction of endothelium-enriched fractions for RNA expression analysis was performed as described previously with some modifications.(Sun et al, 2014; Sun et al, 2012) Briefly, excised vessels were immediately transferred to ice cold PBS. Aortas were mounted on a 20G needle and secured with suture thread. Gluteal vessels had a single end mounted in a DMT 204CM culture myograph chamber. The lumen was gently flushed with ice-cold PBS for 10 seconds. The lumen was then flushed with QIAzol Lysis Reagent (Qiagen Cat.# 79306) for 10 seconds, followed by a 10 second pause before another 10 second elution. Approximately 400 µl of elute was collected from aortas and 50 µl from gluteal vessels. Vessel remainders were then transferred to an empty 1.7 ml tube. To both the vessel remainder and the eluate, 1 ml of TRIzol was immediately added. Vessel remainders were homogenized in a Rescht TissueLyser II set to 30 shakes per second for 6 minutes. RNA was then extracted following the TRIzol protocol.

Appendix Table S1: Demographic, clinical, and medication information and *in vivo* vascular function for the 38 subjects with resistance arterioles that underwent miRNA-Seq profiling

	Non-DM (N=20)	Diabetic (N=18)	P Value
**Age (years)**	51±11	57±8	0.07
**Sex (# female)**	11	8	0.51
**Smoking Status (# never)**	15	9	0.11
**History of Hypertension**	0	15	<0.001
**History of High Cholesterol**	0	11	<0.001
**Body Mass Index (kg/m2)**	26.5±4.2	37.5±7.6	<0.001
**Waste Circumference (cm)**	92.0±9.9	118.8±15.7	<0.001
**Systolic Blood Pressure (mmHg)**	119±12	135±18	0.002
**Diastolic Blood Pressure (mmHg)**	71±7	73±11	0.42
**Fasting Glucose (mg/dL)**	81±12	129±46	<0.001
**Hemoglobin A1C (%)**	5.3±0.5	7.1±1.6	<0.001
**Creatinine (mg/dL)**	0.91±0.18	0.88±0.16	0.62
**Total Cholesterol (mg/dL)**	195±27	165±39	0.009
**HDL Cholesterol (mg/dL)**	60±14	51±12	0.046
**LDL Cholesterol (mg/dL)**	114±25	89±37	0.018
**Medications (% on Therapy)**			
Biguanide	0	55.6	
Sulfonylurea	0	33.3	
Thiazoladinedione	0	0	
DPP4 Inhibitor	0	5.6	
Insulin	0	5.6	
HMG CoA-Reductase Inhibitor	0	38.9	
ACE Inhibitor	0	38.9	
Angiotensin-II Receptor Blocker	0	5.6	
**Brachial Artery Measurements**			
Baseline Diameter (mm)	3.72±0.55	3.75±0.79	0.87
Absolute Flow Mediated Dilation (%)	0.21±0.12	0.16±0.10	0.17
Percent Flow Mediated Dilation (mm)	5.7±3.0	4.4±2.8	0.27
Resting Flow (mL/min)	63±19	90±48	0.035
Peak Flow (ml/min)	418±127	424±215	0.91
Nitroglycerin Mediated Vasodilation (%)	22.2±6.9	16.9±6.9	0.15

Appendix Table S2. Known human miRNAs in the top 30 miRNAs most significantly differentially expressed in gluteal arterioles of DM compared to Non-DM subjects. The miRNAs shown in bold have been reported to exhibit changes in their plasma levels in DM patients.

name	p.values.gps	non-DM read count	DM read count	log_2FoldChange (normalized)
hsa-let-7a-3p	0.0002	4±1	19±3	2.06
hsa-let-7i-3p	0.0005	2±1	11±3	2.24
hsa-miR-29c-3p	0.0031	55±10	112±14	1.02
hsa-miR-146b-5p	0.0034	139±26	458±127	1.73
hsa-miR-146b-3p	0.0041	5±2	18±4	1.74
hsa-miR-212-3p	0.0042	1±0	5±1	2.08
**hsa-miR-29a-3p**	0.0053	199±37	409±66	1.04
**hsa-miR-21-5p**	0.0070	644±121	1,675±323	1.38
hsa-miR-28-5p	0.0095	157±29	320±61	1.03
hsa-miR-497-5p	0.0096	76±15	159±26	1.06
hsa-miR-1307-5p	0.0097	24±5	68±15	1.52
**hsa-miR-195-3p**	0.0099	24±6	48±10	1.02
**hsa-miR-99b-3p**	0.0110	8±2	18±3	1.09
**hsa-miR-140-5p**	0.0112	15±5	35±6	1.23
**hsa-miR-126-3p**	0.0117	839±185	1,582±240	0.92
hsa-miR-3607-5p	0.0140	9±2	24±5	1.47
hsa-miR-10a-3p	0.0146	2±1	5±1	1.58
**hsa-miR-29b-3p**	0.0153	4±1	12±2	1.43

Appendix Table S3: Demographic, clinical, and medication information for the 70 subjects with resistance arterioles used in miRNA mimic or anti-miR treatment experiments.

	Non-DM (N=36)	Diabetic (N=34)	P Value
**Age (years)**	40±14	60±9	<0.0001
**Sex (# female)**	17	21	0.58
**Smoking Status (# never)**	28	18	0.47
**History of Hypertension**	0	25	<0.0001
**History of High Cholesterol**	0	19	<0.0001
**Body Mass Index (kg/m2)**	25.7±5.2	33.9±7.7	<0.0001
**Waste Circumference (cm)**	87.9±11.6	114.8±18.4	<0.0001
**Systolic Blood Pressure (mmHg)**	118±11	133±16	<0.0001
**Diastolic Blood Pressure (mmHg)**	68±9	73±10	0.04
**Fasting Glucose (mg/dL)**	81±13	148±43	<0.0001
**Hemoglobin A1C (%)**	5.2±0.5	7.1±1.1	<0.0001
**Creatinine (mg/dL)**	0.80±0.20	0.92±0.20	0.63
**Total Cholesterol (mg/dL)**	190±42	161±31	<0.0001
**HDL Cholesterol (mg/dL)**	68±28	49±10	0.0001
**LDL Cholesterol (mg/dL)**	106±31	78±25	0.0001
**Medications (% on Therapy)**			
Biguanide	0	79	
Sulfonylurea	0	32	
Thiazoladinedione	0	0	
DPP4 Inhibitor	0	12	
Insulin	0	15	
HMG CoA-Reductase Inhibitor	0	59	
ACE Inhibitor	0	38	
Angiotensin-II Receptor Blocker	0	12	

Appendix Table S4. Exact p values. An exact p value was not generated when it was less than 0.001 for some of the tests.

Figure	Note	p value		Figure	Note	p value		Figure	Note	p value
1A	miR-29a-3p	0.0053		2F	2-way ANOVA +/- vs. +/+	0.028		5A		0.0223
	miR-29b-3p	0.0153			2-way ANOVA -/- vs. +/-	0.001		5B	DAF2	0.0494
1C	2-way ANOVA	<0.001			Ach -6 +/- vs. +/+	0.017			Nitrite/nitrate	0.0003
	Ach -8	<0.001			Ach -5 +/- vs. +/+	0.043		5C	DAF2	0.0193
	Ach -7	<0.001			Ach -6 -/- vs. +/+	0.005			Nitrite/nitrate	0.0038
	Ach -6	<0.001			Ach -5 -/- vs. +/+	<0.001		5D	DAF2	0.0012
	Ach -5	<0.001			Ach -6 -/- vs. +/-	0.01			Nitrite/nitrate	0.002
1D	2-way ANOVA	<0.001			Ach -5 -/- vs. +/-	0.008		5E		0.0114
	Ach -9	0.04		2G	Day 4	0.017		5F		0.008
	Ach -8	0.001			Day 5	0.008		5G	2-way ANOVA	<0.001
	Ach -7	<0.001			Day 6	0.003			Ach -7	0.012
	Ach -6	<0.001			Day 7	0.004			Ach -6	0.001
	Ach -5	<0.001			Day 8	0.002			Ach -5	<0.001
2A	2-way ANOVA	< 0.001			Day 9	0.002		5H		0.0137
	Ach -7	<0.001			Day 10	<0.001		5I	2-way ANOVA	0.004
	Ach -6	<0.001			Day 11	0.004			Ach -7	0.005
	Ach -5	<0.001			Day 12	0.005			Ach -6	<0.001
2B	2-way ANOVA anti-miR-29a	<0.001			Day 13	0.034			Ach -5	<0.001
	2-way ANOVA anti-miR-29b	<0.001			Day 14	0.014		5J	2-way ANOVA	<0.001
	Ach -7 anti-miR-29a	<0.001		3A	scr. Mimic	0.001			Ach -8	<0.001
	Ach -6 anti-miR-29a	<0.001			scr. Mimic+L-NAME	<0.001			Ach -7	<0.001
	Ach -5 anti-miR-29a	<0.001			29b mimic+L-NAME	0.003			Ach -6	<0.001
	Ach -7 anti-miR-29b	<0.001		3B		0.03			Ach -5	<0.001
	Ach -6 anti-miR-29b	<0.001		3C	+/-	<0.001		S1	146b-5p	0.04
	Ach -5 anti-miR-29b	<0.001			-/-	<0.001			29a-3p	0.045
2D	PCAM-1	0.036		3D	2-way ANOVA	<0.001			29b-3p	0.046
	MYH11	<0.001			Ach -8	0.003		S2A		0.047
2E	29b-3p +/- vs. +/+	0.004			Ach -7	<0.001		S2B		0.045
	29b-3p -/- vs. +/+	<0.001			Ach -6	<0.001		S4A	PCAM-1	0.0464
	29b-3p -/- vs. +/-	0.014			Ach -5	<0.001			MYH11	0.0133
	29a-3p +/- vs. +/+	0.005		4B		<10^-30		S4B		0.0057
	29a-3p -/- vs. +/+	<0.001		4C		0.0016		S8		0.0391
				4E	human	0.0041				
					rat	<0.001				

Appendix Figure S1. Real-time PCR verification of small RNA deep sequencing results. Real-time PCR analysis was performed for three miRNAs using the original, individual RNA samples.

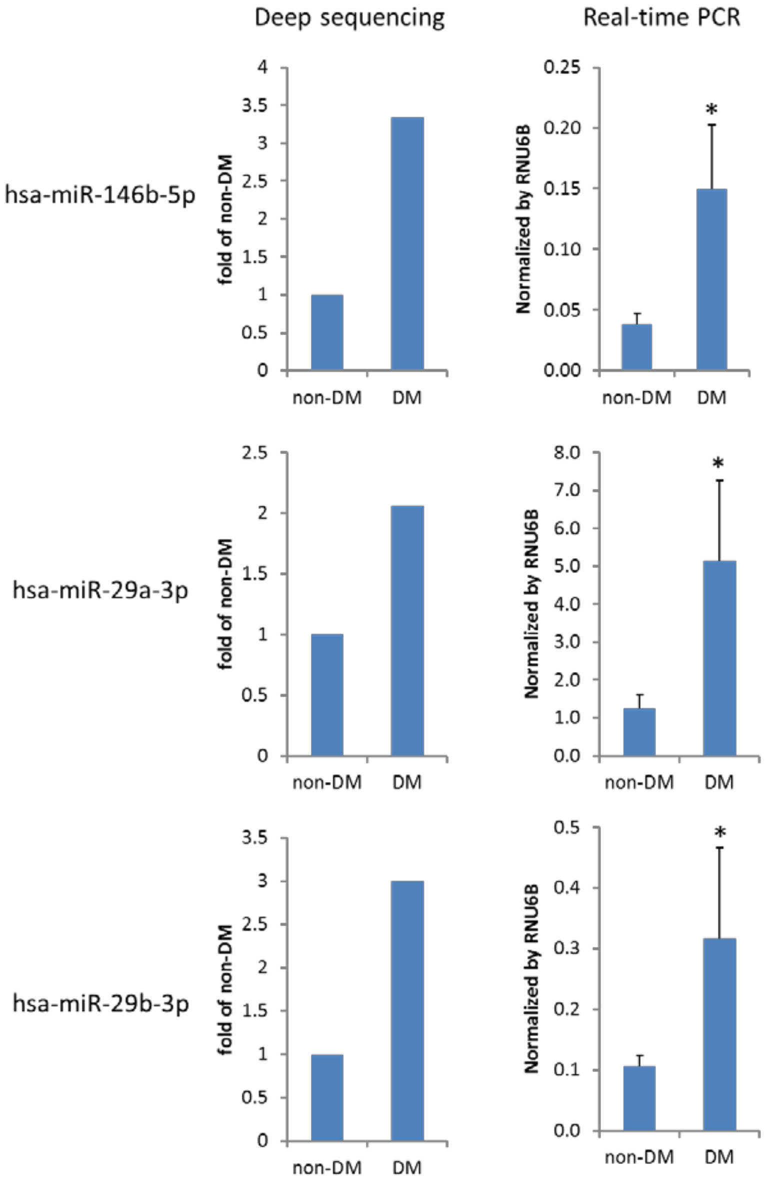

Appendix Figure S2. Confirmation of miR-29 mimic transfection in human gluteal arterioles. miR-29a-3p (**A**) and miR-29b-3p (**B**) abundance was analyzed with real-time PCR in human gluteal arterioles that had been used for the endothelium-dependent vasodilation experiments described in Figure 1D and 1E. The vessels had been transfected intra-luminally with scrambled, miR-29a-3p, or miR-29b-3p mimic for 4 hours followed by approximately 20 hours of perfusion in a culture myograph. The abundance was normalized by 5S rRNA. N=6, *, $p<0.05$ vs. scrambled mimic.

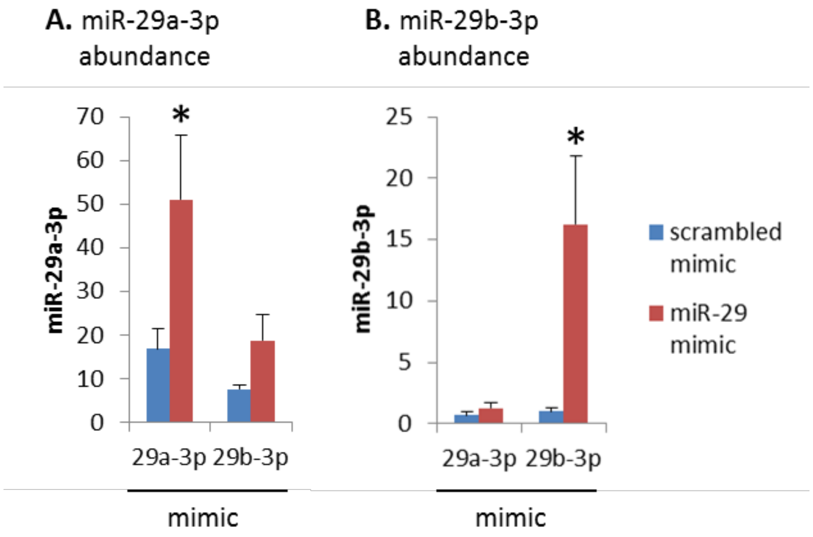

Appendix Figure S3. Spermine NONOate, a nitric oxide (NO) donor, induced robust dilation in human arterioles after intraluminal treatment with anti-miR-29b-3p. N=5, P=0.41.

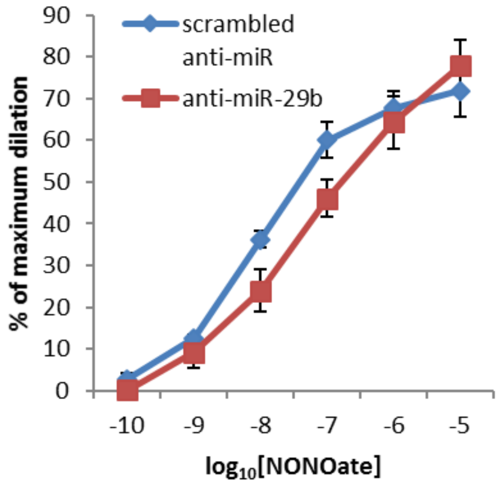

Appendix Figure S4. Abundance of miR-29b-3p in the EC elute from the aorta of *Mir29b-1/a*$^{-/-}$ rats. ***A.*** An endothelium-enriched fraction (EC elute) was extracted from rat aorta. PECAM-1 and MYH11 are marker genes for endothelial and smooth muscle cells, respectively. n=17, *, p<0.05 vs. the remainder of the vessel. ***B.*** Abundance of miR-29b-3p in the EC elute from the aorta. n=6, *, P<0.05, Kruskal-Wallis One Way Analysis of Variance on Ranks due to lack of normality.

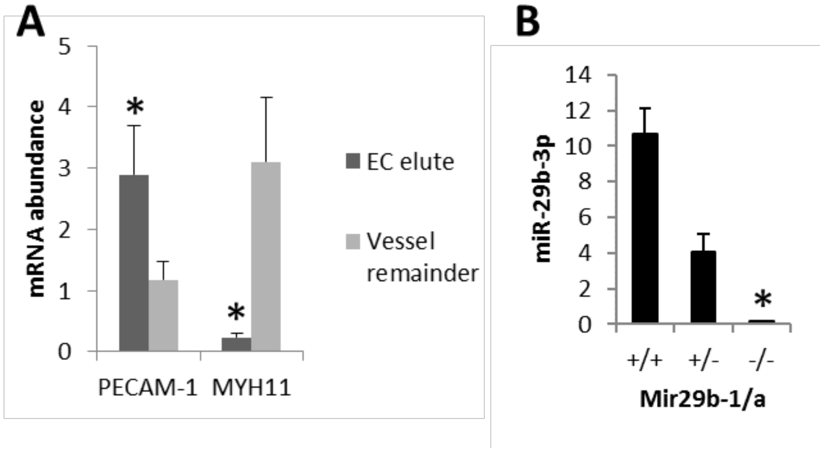

Appendix Figure S5. The NO donor spermine NONOate induced robust dilation in arterioles from *Mir29b-1/a*$^{-/-}$ rats. N=10.

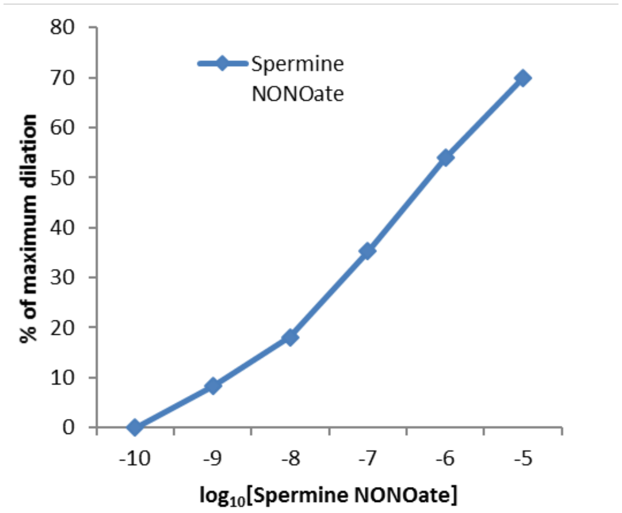

Appendix Figure S6. eNOS protein abundance in mesentery arteries was not changed in *Mir29b-1/a*$^{-/-}$ rats. Primary antibody was BD Transduction, catalog #610296, and used at 1:2500 dilution. Secondary antibody was Sigma, catalog #A9044, and used as 1:10,000 dilution.

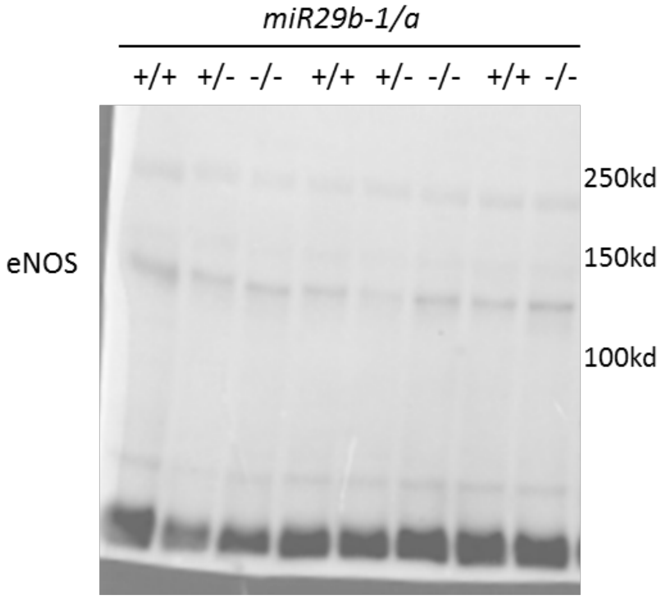

Appendix Figure S7. RNA-seq data from gluteal arterioles correctly clustered *Mir29b-1/a*$^{-/-}$ rats (KO) and wild-type littermates (WT) separately.

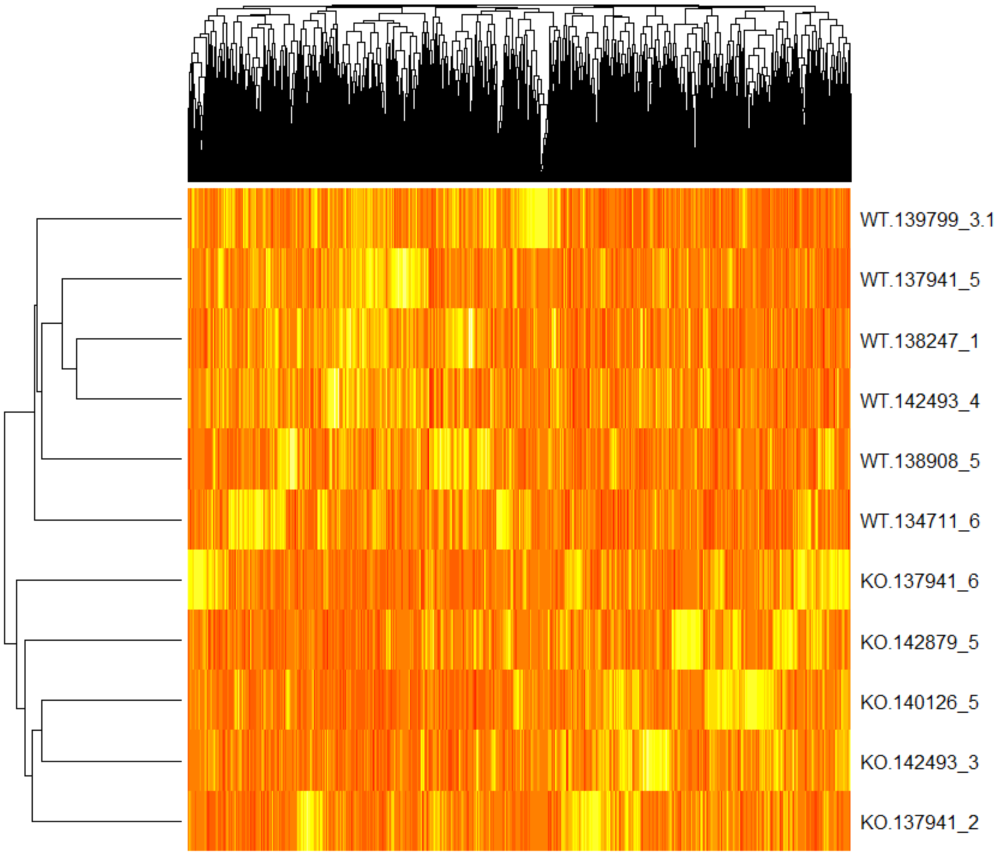

Appendix Figure S8. Abundance of Gap43 mRNA in the carotid artery of *Mir29b-1/a*$^{-/-}$ rats. *Gap43* is a predicted target gene of miR-29 that can reduce eNOS activation. N=5, *, P<0.05, student t-test.

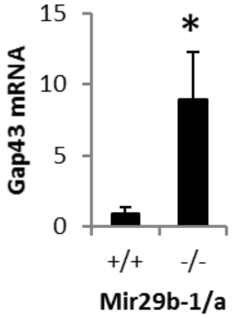

References:

Babar GS, Zidan H, Widlansky ME, Das E, Hoffmann RG, Daoud M, Alemzadeh R (2011) Impaired endothelial function in preadolescent children with type 1 diabetes. Diabetes Care 34**:** 681-685

Dharmashankar K, Welsh A, Wang J, Kizhakekuttu TJ, Ying R, Gutterman DD, Widlansky ME (2012) Nitric oxide synthase-dependent vasodilation of human subcutaneous arterioles correlates with noninvasive measurements of endothelial function. Am J Hypertens 25**:** 528-534

Griendling KK, Touyz RM, Zweier JL, Dikalov S, Chilian W, Chen YR, Harrison DG, Bhatnagar A, American Heart Association Council on Basic Cardiovascular S (2016) Measurement of Reactive Oxygen Species, Reactive Nitrogen Species, and Redox-Dependent Signaling in the Cardiovascular System: A Scientific Statement From the American Heart Association. Circ Res

Kizhakekuttu TJ, Gutterman DD, Phillips SA, Jurva JW, Arthur EI, Das E, Widlansky ME (2010) Measuring FMD in the brachial artery: how important is QRS gating? J Appl Physiol 109**:** 959-965

Kizhakekuttu TJ, Wang J, Dharmashankar K, Ying R, Gutterman DD, Vita JA, Widlansky ME (2012) Adverse alterations in mitochondrial function contribute to type 2 diabetes mellitus-related endothelial dysfunction in humans. Arterioscler Thromb Vasc Biol 32**:** 2531-2539

Kojima H, Nakatsubo N, Kikuchi K, Kawahara S, Kirino Y, Nagoshi H, Hirata Y, Nagano T (1998) Detection and imaging of nitric oxide with novel fluorescent indicators: diaminofluoresceins. Anal Chem 70**:** 2446-2453

Kriegel AJ, Baker MA, Liu Y, Liu P, Cowley AW, Jr., Liang M (2015) Endogenous microRNAs in human microvascular endothelial cells regulate mRNAs encoded by hypertension-related genes. Hypertension 66**:** 793-799

Kriegel AJ, Liu Y, Cohen B, Usa K, Liu Y, Liang M (2012) MiR-382 targeting of kallikrein 5 contributes to renal inner medullary interstitial fibrosis. Physiol Genomics 44**:** 259-267

Kriegel AJ, Liu Y, Liu P, Baker MA, Hodges MR, Hua X, Liang M (2013) Characteristics of microRNAs enriched in specific cell types and primary tissue types in solid organs. Physiol Genomics 45**:** 1144-1156

Liang M, Pietrusz JL (2007) Thiol-related genes in diabetic complications: a novel protective role for endogenous thioredoxin 2. Arterioscler Thromb Vasc Biol 27**:** 77-83

Liu Y, Liu P, Yang C, Cowley AW, Jr., Liang M (2014) Base-resolution maps of 5-methylcytosine and 5-hydroxymethylcytosine in Dahl S rats: effect of salt and genomic sequence. Hypertension 63**:** 827-838

Liu Y, Taylor NE, Lu L, Usa K, Cowley AW, Jr., Ferreri NR, Yeo NC, Liang M (2010) Renal medullary microRNAs in Dahl salt-sensitive rats: miR-29b regulates several collagens and related genes. Hypertension 55**:** 974-982

Mladinov D, Liu Y, Mattson DL, Liang M (2013) MicroRNAs contribute to the maintenance of cell-type-specific physiological characteristics: miR-192 targets Na+/K+-ATPase beta1. Nucleic Acids Res 41**:** 1273-1283

Ou J, Ou Z, Jones DW, Holzhauer S, Hatoum OA, Ackerman AW, Weihrauch DW, Gutterman DD, Guice K, Oldham KT, Hillery CA, Pritchard KA, Jr. (2003) L-4F, an apolipoprotein A-1 mimetic, dramatically improves vasodilation in hypercholesterolemia and sickle cell disease. Circulation 107**:** 2337-2341

Suboc TM, Dharmashankar K, Wang J, Ying R, Couillard A, Tanner MJ, Widlansky ME (2013) Moderate Obesity and Endothelial Dysfunction in Humans: Influence of Gender and Systemic Inflammation. Physiol Rep 1

Sun X, He S, Wara AKM, Icli B, Shvartz E, Tesmenitsky Y, Belkin N, Li D, Blackwell TS, Sukhova GK, Croce K, Feinberg MW (2014) Systemic delivery of microRNA-181b inhibits nuclear factor-kappaB activation, vascular inflammation, and atherosclerosis in apolipoprotein E-deficient mice. Circ Res 114**:** 32-40

Sun X, Icli B, Wara AK, Belkin N, He S, Kobzik L, Hunninghake GM, Vera MP, Blackwell TS, Baron RM, Feinberg MW (2012) MicroRNA-181b regulates NF-kappaB-mediated vascular inflammation. J Clin Invest 122**:** 1973-1990

Tian Z, Greene AS, Pietrusz JL, Matus IR, Liang M (2008a) MicroRNA-target pairs in the rat kidney identified by microRNA microarray, proteomic, and bioinformatic analysis. Genome Res 18**:** 404-411

Tian Z, Greene AS, Usa K, Matus IR, Bauwens J, Pietrusz JL, Cowley AW, Jr., Liang M (2008b) Renal regional proteomes in young Dahl salt-sensitive rats. Hypertension 51**:** 899-904

Tian Z, Liu Y, Usa K, Mladinov D, Fang Y, Ding X, Greene AS, Cowley AW, Jr., Liang M (2009) Novel role of fumarate metabolism in dahl-salt sensitive hypertension. Hypertension 54**:** 255-260

Wang J, Alexanian A, Ying R, Kizhakekuttu TJ, Dharmashankar K, Vasquez-Vivar J, Gutterman DD, Widlansky ME (2012) Acute Exposure to Low Glucose Rapidly Induces Endothelial Dysfunction and Mitochondrial Oxidative Stress: Role for AMP Kinase. Arterioscler Thromb Vasc Biol 32**:** 712-720
